# Cheongsangbangpung-tang ameliorated the acute inflammatory response via the inhibition of NF-κB activation and MAPK phosphorylation

**DOI:** 10.1186/s12906-016-1501-6

**Published:** 2017-01-13

**Authors:** Seon Young Kim, Sang Mi Park, Min Hwangbo, Jong Rok Lee, Sung Hui Byun, Sae Kwang Ku, Il Je Cho, Sang Chan Kim, Seon Young Jee, Sook Jahr Park

**Affiliations:** 1 College of Korean Medicine, Daegu Haany University, 1 Haanydaero, Gyeongsan, 38610 Republic of Korea; 2 Medical Research Center for Globalization of Herbal Formulation, Daegu Haany University, 1 Haanydaero, Gyeongsan, 38610 Republic of Korea; 3 Department of Pharmaceutical Engineering, Daegu Haany University, 1 Haanydaero, Gyeongsan, 38610 Republic of Korea

**Keywords:** Cheongsangbangpung-tang, Inflammation, Nuclear factor-kappaB, Mitogen-activated protein kinase, Paw oedema

## Abstract

**Background:**

Cheongsangbangpung-tang (CBT) is a traditional herbal formula used in Eastern Asia to treat heat-related diseases and swellings in the skin. The present study was conducted to evaluate the anti-inflammatory effects of cheongsangbangpung-tang extract (CBTE) both in vitro and in vivo.

**Methods:**

The in vitro effects of CBTE on the lipopolysaccharide (LPS)-induced production of inflammation-related proteins were examined in RAW 264.7 cells. The levels of nitric oxide (NO) were measured with the Griess reagent. Inflammatory cytokines and prostaglandin E_2_ (PGE_2_) were detected using the enzyme-linked immunosorbent assay (ELISA) method. Inflammation-related proteins were detected by Western blot. The effect of CBTE on acute inflammation in vivo was evaluated using carrageenan (CA)-induced paw oedema. To evaluate the anti-inflammatory effect, paw oedema volume, thickness of the dorsum and ventrum pedis skin, number of infiltrated inflammatory cells, and number of COX-2-, iNOS-immunoreactive cells were measured.

**Results:**

In an in vitro study, CBTE inhibited the production of NO and PGE_2_ and also decreased the expression of inducible nitric oxide synthase (iNOS), cyclooxygenase-2 (COX-2) activity, interleukin (IL)-1β, IL-6 and tumuor necrosis factor-α. In LPS-activated macrophages, nuclear factor-kappaB (NF-κB) and mitogen-activated protein kinase (MAPK) signalling is a pivotal pathway in the inflammatory process. These plausible molecular mechanisms increased the phosphorylation of I-κBα, while the activation of NF-κB and the phosphorylation of MAPK by LPS were blocked by CBTE treatment. In our in vivo study, a CA-induced acute oedematous paw inflammation rat model was used to evaluate the anti-inflammatory effect of CBTE. CBTE significantly reduced the increases in paw swelling, skin thicknesses, infiltrated inflammatory cells and iNOS-, COX-2 positive cells induced by CA injection.

**Conclusions:**

Based on these results, CBTE should favourably inhibit the acute inflammatory response through modulation of NF-κB activation and MAPK phosphorylation. Furthermore, the inhibition of CBTE in rat paw oedema induced by CA is considered to be clear evidence that CBTE may be a useful source to treat inflammation.

## Background

Cheongsangbangpung-tang (CBT; Qing-Shang-Fang-Feng-Tang in Chinese and Seijo-bofu-to in Japanese) is a traditional herbal formulation composed of 13 medicinal herbs: *Ledebouriellae radix*, *Angelicae dahuricae radix*, *Forsythiae fructus*, *Platycodi radix*, *Scutellariae radix*, *Cnidii rhizoma*, *Schizonepetae herba*, *Gardeniae fructus*, *Coptidis rhizoma*, *Aurantii fructus*, *Menthae herba*, *Glycyrrhizae radix*, and *Bambusae caulis* in Liquamen. In Korean medicine, CBT has been used to treat inflammatory diseases, such as conjunctivitis, eczema, acne, furuncles on the face, rosacea, and ophthalmitis [1, 2].

The immune system is one feature found in living organisms that acts as a defence against external pathogenic substances, such as bacteria, viruses and fungi. Once infected with pathogenic microorganisms, the skin and mucosal surfaces serve as primary physical barriers [3]. An immune response (immunity) is launched to resist infection when a physical barrier has been compromised [4, 5]. Homeostasis of the human body can be maintained with proper control of the immune response. In addition, an abnormal or overactive immune system can cause pathogenic conditions, such as asthma, autoimmunity and hypersensitivity [6, 7].

Inflammation is one local defence reaction that is vital for protecting living tissue from injury. However, inflammation may have a negative impact on health if it is extreme or continuously repeats [8]. Chronic inflammation can cause a variety of conditions, such as rheumatoid arthritis, cardiovascular disease and cancer [9]. Various substances, such as cytokines and prostaglandins (PGs), are involved in the inflammatory response. Lipopolysaccharides (LPS) from gram-negative bacteria can trigger sepsis by activating macrophage cells and leading to the abnormal generation of cytokines and PGs [10, 11]. Therefore, macrophage cells that have been stimulated by LPS present a good model system for studying the anti-inflammatory activities of potent candidate materials. In traditional Korean medicine, many herbal formulas have been prescribed to treat inflammatory diseases [12]. CBT is expected to have anti-inflammatory effects in both in vitro and in vivo studies because it traditionally has been used to bring down the fever [1, 2]. However, the mechanism of action of the anti-inflammatory activity of CBT has not yet been reported.

In this study, we evaluated the anti-inflammatory effects of CBT extract (CBTE) on both animal and cellular inflammation models. An LPS-inducible macrophage was used for the in vitro investigation of anti-inflammation. In addition, a carrageenan (CA)-induced paw oedema experiment was conducted to determine the effect on the acute-phase inflammation in vivo.

## Methods

### Preparation of the aqueous cheongsangbangpung-tang extract

CBT is composed of 13 herbs (Table 1), which were purchased from Daewon Pharmacy (Daegu, Korea). Ten folds the amount of CBT (284.6 g) of 12 herbs except *Bambusae caulis* in Liquamen were extracted by boiling in 2 l of water for 3 h and then adding 187.5 g of *Bambusae caulis* in Liquamen to the hot water extract; the solution was placed into a cotton bag and squeezed to obtain the final aqueous extract of CBT (CBTE), which was filtered through 0.2-μm filter paper (Nalgene, NY, USA), lyophilized and stored at −20 °C until its use.

**Table 1:** Composition of Cheongsangbangpung-tang

Herbal scientific name	Amount (g)	Herbal scientific name	Amount (g)
*Ledebouriellae radix*	3.75	*Gardeniae fructus*	1.87
*Angelicae Dahuricae radix*	3.0	*Coptidis rhizoma*	1.87
*Forsythiae fructus*	3.0	*Aurantii fructus*	1.87
*Platycodi radix*	3.0	*Menthae herba*	1.87
*Scutellariae radix*	2.62	*Glycyrrhizae radix*	1.12
*Cnidii rhizoma*	2.62	*Bambusae caulis in Liquamen*	18.75
*Schizonepetae herba*	1.87		

### Reagents

The LPS (*E. coli* 026:B6) and Griess reagent were obtained from Sigma (St. Louis, MO, USA). Glycyrrhizic acid, liquiritigenin, berberine, baicalin, baicalein, forsythiaside-A and poncirin were also purchased from Sigma. Enzyme-linked immunosorbent assay (ELISA) kits for tumour necrosis factor-alpha (TNF-α), interleukin (IL)-6 and IL-1β were acquired from Pierce Endogen (Thermo Scientific, Waltham, MA, USA). The PGE_2_ ELISA kit was obtained from R&D Systems (Minneapolis, MN, USA). The primary antibodies for the phospho (p)-inhibitor of NF-κB α (I-κBα), p-ERK, p-jun NH_2_-terminal kinase (JNK), p-p38, ERK, JNK and p38 antibodies were purchased from Cell Signaling (Danvers, MA, USA). COX-2 and iNOS antibodies were obtained from BD Biosciences (San Jose, CA, USA), while the NF-κB, IκBα, β-actin and lamin A/C antibodies were purchased from Santa Cruz Biotechnology (Dallas, TX, USA).

### Chromatographic analysis of CBTE

An ultra performance liquid chromatography (UPLC) system (Waters, USA) was used to identify the seven marker phytochemicals of CBTE: glycyrrhizic acid, liquiritigenin, berberine, baicalin, baicalein, forsythiaside-A, and poncirin. The system was equipped with an ACQUITYTM photodiode array detector (PDA) and a binary solvent delivery pump. The separation was executed on an ACQUITYTM BEH Shield RP 18 column (1.7 μm, 2.1 × 100 mm). The mobile phase was composed of 0.1% formic acid in water (solvent A) and 0.1% formic acid in acetonitrile (solvent B) with a gradient elution system at a flow rate of 1 ml/min. The injection volume was 2 μl. The column temperature was set at room temperature.

### Cell culture and cell viability test

The RAW264.7 macrophage cell line was obtained from American Type Culture Collection (Manassas, VA, USA) and maintained in Dulbecco’s modified Eagle’s medium containing 10% foetal bovine serum made quiescent by serum starvation for 12 h. For all experiments, LPS was used at a final concentration of 1 μg/ml. Test samples were added to the culture medium 1 h prior to the addition of LPS and treated for the indicated time periods. For the cell viability experiment, the cells were stained with 3-(4,5-dimethylthiazol-2-yl)-2,5-diphenyltetrazolium bromide (MTT) solution (0.5 mg/ml) for 4 h. The dark blue crystals of formazan were dissolved into 200 μl of dimethyl sulphoxide, and the absorbance of the formazan solution was measured at 570 nm.

### Determination of NO, PGE_2_ and cytokines

NO production was monitored by measuring the nitrite content in the culture media by mixing the cultured media with Griess reagent (1% sulphanilamide, 0.1% *N*-1-naphthylenediamine dihydrochloride and 2.5% phosphoric acid). The absorbance was measured at 540 nm after 10 min of incubation.

The levels of PGE_2_ and cytokines (TNF-α, IL-1β, and IL-6) were assessed by ELISA using anti-mouse PGE_2_, TNF- α, IL-1 β, or IL-6 antibodies and biotinylated secondary antibody according to the manufacturer’s instructions.

### Assay of COX-2 activity

COX-2 enzyme activity was measured using an ELISA kit (Cayman Chemical Co., Ann Arbor, MI, USA). The cells were collected by centrifugation at 1500×*g* for 10 min at 4 °C and homogenized in a cold buffer (0.1 M Tris-HCl, pH: 7.8, 1 mM EDTA, 250 mM mannitol, and 0.3 mM diethyldithiocarbamic acid). The homogenized cells were centrifuged at 10,000×*g* for 15 min at 4 °C, and the supernatant (cell lysate) was removed for the COX-2 activity assay according to the manufacturer’s instructions.

### Western blot analysis

Whole-cell lysates were prepared by lysing cells in an RIPA buffer, and the nuclear fractions for the NF-κB analysis were set up using a commercial kit (Chemicon International, Inc., Billerica, MA, USA). Equal amounts of proteins were resolved on 10% sodium dodecyl sulphate gel and then transferred to a nitrocellulose membrane. The membrane was incubated with primary antibodies for 2 h, followed by incubation with a secondary antibody for 1 h. The immunoreactivity was detected using a chemiluminescent detection kit (Amersham Bioscience, Piscataway, NJ, USA).

### Statistical analysis for cell experiments

Statistical evaluations were performed using the Statistical Package for the Social Sciences (SPSS) software (version 18, IBM Corp., Armonk, NY, USA). A one-way analysis of variance followed by a Tukey’s test was used to assess the differences among treatment groups. Values are expressed as mean ± S.D. The criterion for statistical significance was set at *P* < 0.05 or *P* < 0.01.

### Carrageenan-induced paw oedema

Sprague-Dawley rats at 6 weeks of age (male, 140–160 g) were provided by the Samtako Co. (Osan, Korea), allowed to acclimate to their new environment for 1 week and maintained in a clean room at the animal lab. The animals were caged under the supply of filtered, pathogen-free air and fed standard rat chow (Purina, Korea) and water *ad libitum* at a temperature between 20 and 23 °C with 12 h light and dark cycles and a relative humidity of 50%. All animal experiments were conducted in strict accordance with protocols approved by the Institutional Animal Care and Use Committee of Daegu Haany University (Approval number: DHU 2015-064). The rats were divided into five groups: normal; CA control; CA + dexamethasone (DEXA, 1 mg/kg) as a standard reference; CA + CBTE (0.3 g/kg); and CA + CBTE (1.0 g/kg). CBTE and DEXA were dissolved in saline and orally administered to the control rats for four consecutive days. Paw oedema was induced by injecting 0.1 ml of 1% w/v CA suspended in saline into the sub-plantar tissues of the right hind paw of each rat. The volume of the paw was measured with a Plethysmometer (LE 7500; LETICA Scientific Instruments, Spain) immediately prior to the CA injection and then again 1, 2, 3 and 4 h after the injection. The data were expressed as the variation in the paw volume (ml) and were compared to the pre-injection values.

### Histological process

The dorsum and ventrum pedis skins of the hind paws were separated and fixed in 10% neutral buffered formalin then embedded in paraffin, sectioned (3 ~ 4 μm) and stained with haematoxylin and eosin (HE) to determine their general histopathological profiles and the immunohistochemistry for COX-2 and iNOS.

### Immunohistochemistry

The changes in the immunoreactivities of COX-2 and iNOS were observed by purified primary antibodies with avidin-biotin-peroxidase (ABC) and peroxidase substrate kits (Vector Labs, Burlingame, CA, USA). Briefly, the endogenous peroxidase activity was blocked by incubation in methanol and 0.3% H_2_O_2_ for 30 min, while non-specific binding of immunoglobulin was blocked with normal horse serum blocking solution for 1 h in a humidity chamber after heating (95–100 °C)-based epitope retrievals in 10 mM citrate buffers (pH: 6.0). The primary antisera were treated overnight at 4 °C in a humidity chamber and then incubated with biotinylated universal secondary antibody and ABC reagents for 1 h at room temperature in a humidity chamber. Finally, the sections were reacted with a peroxidase substrate kit for 3 min at room temperature.

### Histomorphometry

To observe more detailed changes induced by CA either with or without CBTE treatment, the thicknesses of the dorsum and ventrum pedis skins (from the epidermis to the dermis; keratin layers were excluded) were measured with an automated image analyzer (iSolution FL ver 9.1, IMT i-solution Inc., Vancouver, Quebec, Canada) and microscopy at 40× magnification (Model Eclipse 80i, Nikon, Tokyo, Japan) to prepare skin histological samples as μm/paw; the number of infiltrated inflammatory cells in the dermis was also counted using an automated image analyser as cells/mm^2^ of dermis under 200× magnification. In addition, the cells occupied by over 10% of COX-2 and iNOS immunoreactivities were regarded as positive. In the present study, the number of COX-2- and iNOS-positive cells were separately calculated on the epithelial lining as cells/100 epithelial cells and on the mm^2^ of dermis (cells/mm^2^ of dermis) using a digital image analyser. The histopathologist was blinded to the group distribution when this analysis was carried out.

### Statistical analysis for animal experiments

Multiple comparison tests for different dose groups were conducted. The variance homogeneity was examined using the Levene test. If the Levene test indicated no significant deviations from the variance homogeneity, the obtained data were analysed by a one-way analysis of variance (ANOVA) test followed by a least-significant differences (LSD) multi-comparison test to determine which pairs of group comparisons were significantly different. Differences were considered significant at *P* < 0.05. In addition, the per cent-point changes between normal and CA groups were calculated to monitor the severities of acute inflammations induced in this study, and the per cent-point changes between CA and test material, CBTE or DEXA-treated skins were also determined to further the understanding of the efficacy as follows: Per cent-point Changes Compared with Normal (%) = ((Data of CA–Data of Normal)/Data of Normal) ×100; and Per cent-point Changes Compared with CA (%) = {(Data of test material treated rats-Data of CA)/Data of CA} ×100.

## Results

### UPLC analysis of CBTE

To conduct a quantitative UPLC analysis of CBTE, we chose five plants for the quality control of Cheongsangbangpung-tang by referring to the Korean Pharmacopoeia and announcement of KFDA (Korean Food and Drug Administration). The Korean Pharmacopoeia was recorded reference standards forsythiaside-A from Forsythiae Fructus, baicalin and baicalein from Scutellariae Radix, berberine from Coptidis Rhizoma, poncirin from Aurantii Fructus, and glycyrrhizic acid and liquiritigenin from Glycyrrhizae Radix [13].

**Fig. 1:**
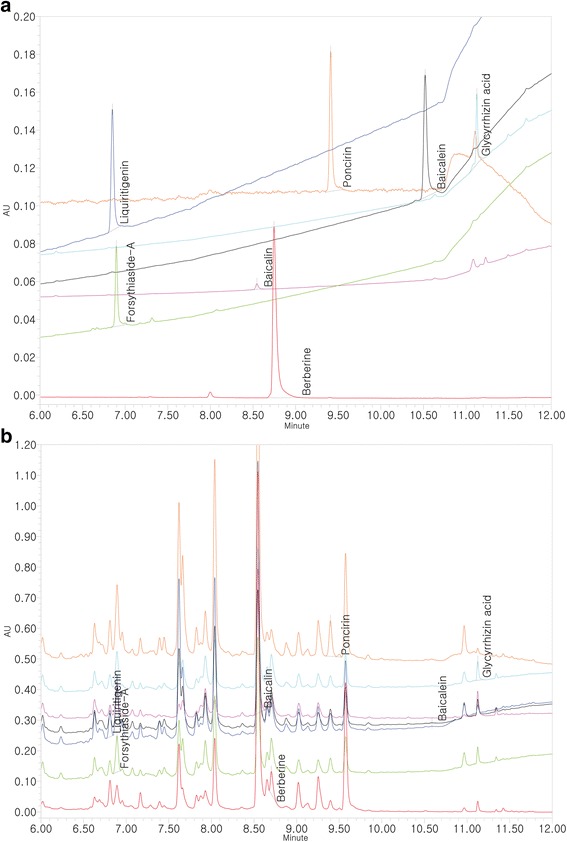
Analysis of seven standard compounds in CBTE by UPLC-PDA. Ultra performance liquid chromatography (UPLC) chromatograms of seven standard compounds: glycyrrhizic acid, liquiritigenin, berberine, baicalin, baicalein, forsythiaside-A and poncirin **a** UPLC chromatograms of glycyrrhizic acid, liquiritigenin, berberine, baicalin, baicalein, forsythiaside-A and poncirin in CBTE (glycyrrhizic acid and liquiritigenin; 254 nm, berberine; 345 nm, baicalin and baicalein; 277 nm, forsythiaside-A; 280 nm, poncirin; 230 nm) **b** CBTE, Cheongsangbangpung-tang extract

The contents of the seven compounds were calculated from the calibration curve of the standard (Fig. 1). All standard peaks were identified within 6 to 12 min of retention time. Two close peaks, liquiritigenin and forsythiaside-A, were displayed at near 7 min of retention time. To avoid confusion of peak identification, liquiritigenin and forsythiaside-A were analyzed using different wavelengths at 254 nm and 280 nm, respectively. The amounts of each compound were as follows: glycyrrhizic acid, 41.59 ± 2.07 ppm; liquiritigenin, 6.72 ± 0.14 ppm; berberine, 7.11 ± 0.37 ppm; baicalin, 4027.47 ± 98.61 ppm; baicalein, 1.95 ± 0.03 ppm; forsythiaside-A, 38.75 ± 0.31 ppm; and poncirin, 10.13 ± 1.44 ppm.

### Effects of CBTE on the creation of NO and the expression of iNOS

**Fig. 2:**
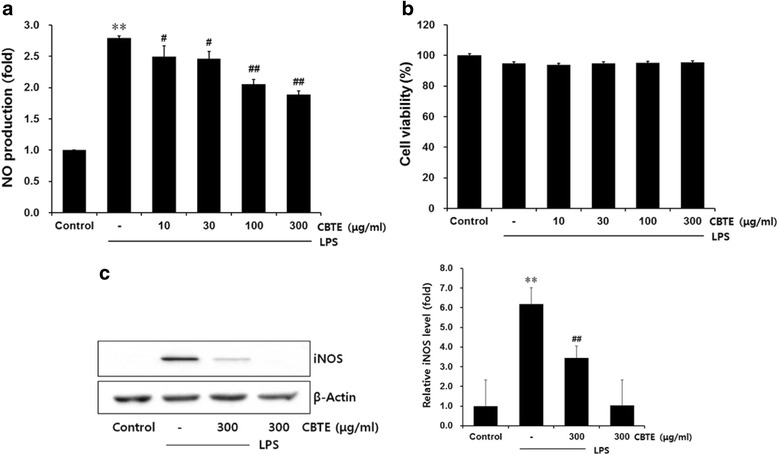
The inhibitory effects of CBTE on NO production and iNOS expression. Raw 264.7 cells were treated with 10–300 μg/ml of CBTE for 1 h prior to the addition of lipopolysaccharide (LPS) (1 μg/ml), and further incubated for 12 h. Cell viability was measured by 3-(4,5-dimethylthiazol-2-yl)-2,5-diphenyltetrazolium bromide assay. The concentrations of nitrite in the culture medium were assessed using the Griess reagent as described in the Methods section (**a**). The effect of CBTE plus LPS on cell viability was determined after 12 h of incubation (**b**). Expression of the iNOS protein was assessed by immunoblotting using a specific anti-iNOS antibody (**c**). Equal amounts of the total protein (50 μg/lane) were separated by SDS-PAGE. β-actin was used as a loading control, and the relative levels of protein bands were measured by scanning densitometry. Values represent the mean ± S.D. of three independent experiments (significant compared to the control, **P < 0.01, significant as compared to LPS alone, ^##^ *P* < 0.01). CBTE, Cheongsangbangpung-tang extract

To evaluate the effects of CBTE on LPS-inducible NO production, the culture medium was exposed to 1 μg/ml of LPS in the presence or absence of CBTE for 12 h and then analysed using a Griess reagent. In the present study, CBTE showed significant inhibitory effects on the elevated synthesis of NO by LPS (Fig. 2a). The levels of NO were decreased by CBTE in a concentration-dependent manner. The cell viability was assessed at different CBTE concentrations to determine if the reduction in NO contributed to the decrease in the overall cell population. During a 12-h incubation with CBTE and LPS, the cell viability was not affected by CBTE at the concentrations used (Fig. 2b). These data indicated that the inhibition of LPS-stimulated NO creation by CBTE was not the result of a cytotoxic effect on these cells.

iNOS is a key enzyme responsible for NO synthesis. Thus, Western blot analysis was performed to determine whether the reduction of NO was related to the modulated expressions of the iNOS protein. LPS (1 μg/ml) strongly induced iNOS expression, whereas there was no detectable band of iNOS protein in the control cells. Pre-treatment with CBTE significantly inhibited iNOS expression, and this expression level was in parallel with the comparable inhibition of NO release (Fig. 2c).

### The effect of CBTE on PGE_2_ production and COX-2 activity

**Fig. 3:**
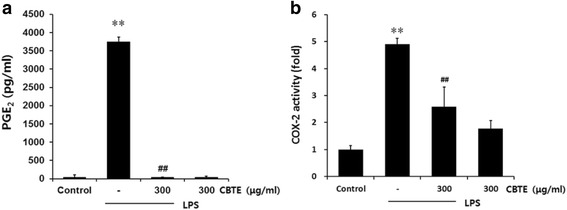
The inhibitory effect of CBTE on the LPS-induced PGE_2_ secretion and COX-2 activity. RAW264.7 cells were treated with CBTE for 1 h prior to the addition of LPS (1 μg/ml), and the cells were further incubated for 12 h. The levels of PGE_2_ in the culture medium were measured with an assay kit as described in the Methods section (**a**). COX-2 enzyme activity was analysed using an assay kit also as described in the Methods section (**b**). Values represent the mean ± S.D. of three independent experiments (significant compared to the control, **P < 0.01, significant as compared to LPS alone, ^##^ *P* < 0.01). CBTE, Cheongsangbangpung-tang extract; PGE_2_, prostaglandin E_2_; COX-2, cyclooxygenase-2

PGE_2_ is a potent mediator of cancer and inflammation. LPS has been shown to increase PGE_2_ production in macrophage cells. In the present study, LPS also caused an immoderate increase of PGE_2_. However, treatment with CBTE markedly decreased the production of PGE_2_ (Fig. 3a). Next, we monitored the effects of CBTE on COX-2 enzyme activity and found that CBTE caused a remarkable inhibition of COX-2 activity, which was greatly enhanced in response to LPS (Fig. 3b). These results suggest that CBTE can inhibit PGE_2_ synthesis by interfering with the enzymatic action of COX-2.

### Reduction of inflammatory cytokines by CBTE

**Fig. 4:**
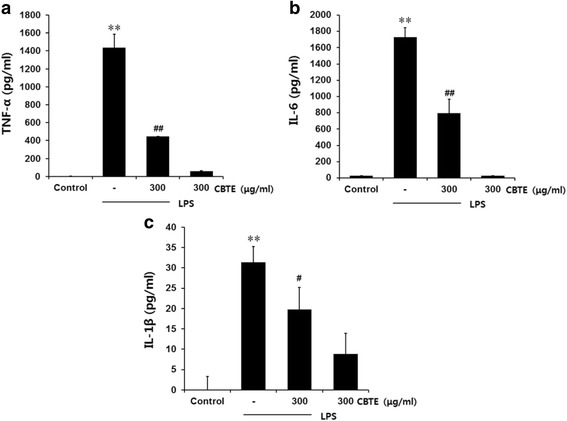
The inhibitory effect of CBTE on the LPS-induced secretion of pro-inflammatory cytokines. RAW264.7 cells were treated with CBTE for 1 h prior to the addition of LPS (1 μg/ml), and the cells were further incubated for 12 h. The concentrations of TNF-α (**a**) IL-6 (**b**) and IL-1β (**c**) in the culture medium were measured using an assay kit as detailed in the Methods section. Values represent the mean ± S.D. of three independent experiments (significant compared to the control, **P < 0.01, significant as compared to LPS alone, ^##^ *P* < 0.01). CBTE, Cheongsangbangpung-tang extract; TNF, tumor necrosis factor; IL, interleukin

We examined whether CBTE displays anti-inflammatory activity by eliminating inflammatory cytokines (TNF-α, IL-1β and IL-6). The culture medium was collected, and ELISA was used to determine the levels of pro-inflammatory cytokines. Our results showed that CBTE significantly (*P* < 0.01) inhibited the augmented synthesis of TNF-α, IL-1β and IL-6 by LPS (Fig. 4).

### Down-regulation of NF-κB signalling by CBTE

**Fig. 5:**
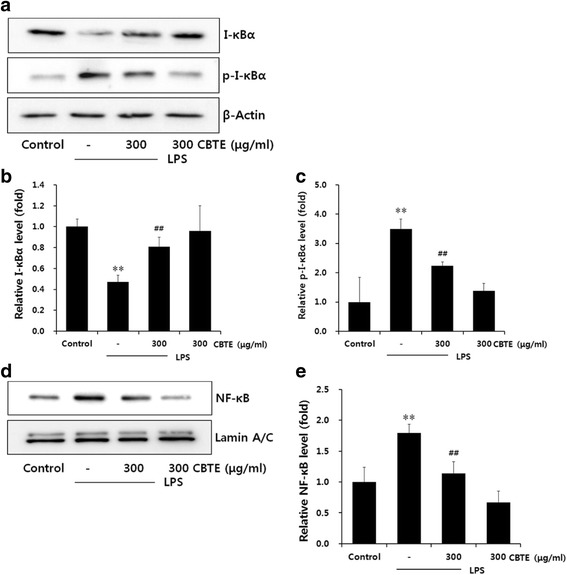
The inhibitory effect of CBTE on the LPS-induced nuclear factor-kappa B protein expressions in RAW264.7 cells. The levels of I-κBα, p-I-κBα (30 min in the cytosol fraction) and NF-κB (1 h in the nuclear fraction) protein were monitored with or without CBTE pre-treatment (i.e. 1 h before LPS). β-actin and lamin A/C were used as a loading control (**a** and **d**). The relative levels of I-κBα (**b**) p-I-κBα (**c**) and NF-κB (**e**) were measured by scanning densitometry. Values represent the mean ± S.D. of three independent experiments (significant as compared to the control, **P < 0.01, significant as compared to LPS alone, ^##^ *P* < 0.01). CBTE, Cheongsangbangpung-tang extract; NF-κB, Nuclear factor-kappa B; I-κB, Inhibitor-kappa B

NF-κB is a key transcription factor that elicits a wide range of biological effects associated with inflammation. Thus, we examined the effect of CBTE on the nuclear translocation of NF-κB via the phosphorylation and degradation of I-κBα. As shown in Fig. 5, the protein level of phosphorylated I-κBα (p-I-κBα) was increased while that of total I-κBα was decreased after 30 min of LPS treatment. However, pretreatment with CBTE led to a marked decrease in the protein levels of p-I-κBα, implying that CBTE can inhibit I-κBα phosphorylation. Moreover, CBTE increased the expression level of I-κBα protein as compared to LPS alone. These results mean that CBTE down-regulated NF-κB signaling by suppressing I-κBα phosphorylation and by recovering I-κBα protein level.

### The effect of CBTE on the phosphorylation of mitogen-activated protein kinase

**Fig. 6:**
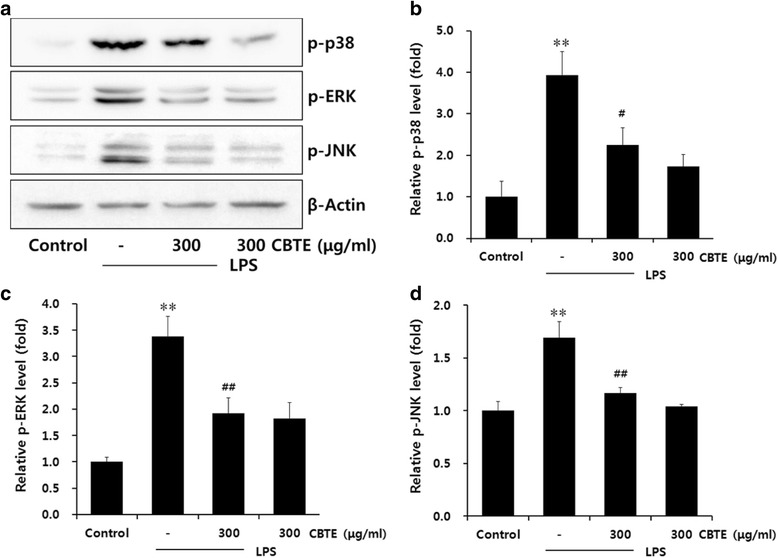
The inhibitory effect of CBTE on the LPS-induced phosphorylation of MAPKs in RAW264.7 cells. The levels of phosphorylation of MAPK proteins were monitored 1 h after treatment of cells with LPS (1 μg/ml) either with or without CBTE pre-treatment (i.e. 1 h before LPS). Expressions of the MAPKs protein were determined by immunoblotting using specific anti-p-p38, anti-p-ERK and anti-p-JNK antibodies. An antibody against β-actin was used to verify equal protein loading of the cell lysate (**a**). The relative levels of the MAPKs were measured by scanning densitometry (**b c d**). Values represent the mean ± S.D. of three independent experiments (significant as compared to the control, **P < 0.01, significant as compared to LPS alone, ^##^ *P* < 0.01). CBTE, Cheongsangbangpung-tang extract; MAPK, Mitogen-activated protein kinases; ERK, extracellular signal-regulated kinase; JNK, Jun N-terminal kinase

Activation of NF-κB signalling is mediated by the upstream mitogen-activated protein kinase (MAPK). Thus, we further investigated the effect of CBTE on the phosphorylation of three MAPKs: ERK, JNK and p38. Based on our results, CBTE significantly suppressed the phosphorylation of MAPK, which was increased by LPS treatment (Fig. 6).

### Effect of CBTE on the formation of CA-induced paw oedema

**Fig. 7:**
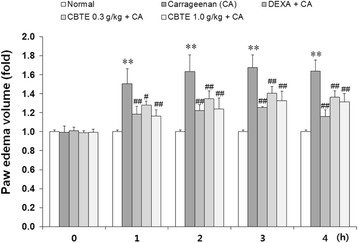
The inhibitory effect of CBTE on carrageenan-induced paw oedema. CBTE was orally administered to rats at 0.3 or 1.0 g/kg/day prior to the induction of paw oedema for 3 days. Paw oedema was induced by the subcutaneous injection of 1% carrageenan solution as described in the Materials and Methods section. The swelling volume of the paw was measured up to 4 h after the carrageenan injection at intervals of 1 h using a plethysmometer. DEXA (1 mg/kg, p.o.) was used as a positive control drug. Values represent the mean ± S.D. of three independent experiments (significant as compared to the control, **P < 0.01, significant as compared to CA alone, ^##^ *P* < 0.01). CBTE, Cheongsangbangpung-tang extract; CA, carrageenan; DEXA, dexamethasone

We used a CA-induced paw oedema model to evaluate the effects of CBTE on acute inflammation in vivo. The inhibition of paw oedema formation by CBTE was calculated using a comparison with CA-treated rats. As expected, the reference drug DEXA (1 mg/kg) caused a significant inhibition of paw oedema. In addition, CBTE significantly (*P* < 0.01) reduced the paw swelling (Fig. 7).

### Histopathological evaluation of CBTE in CA-induced acute inflammation

**Fig. 8:**
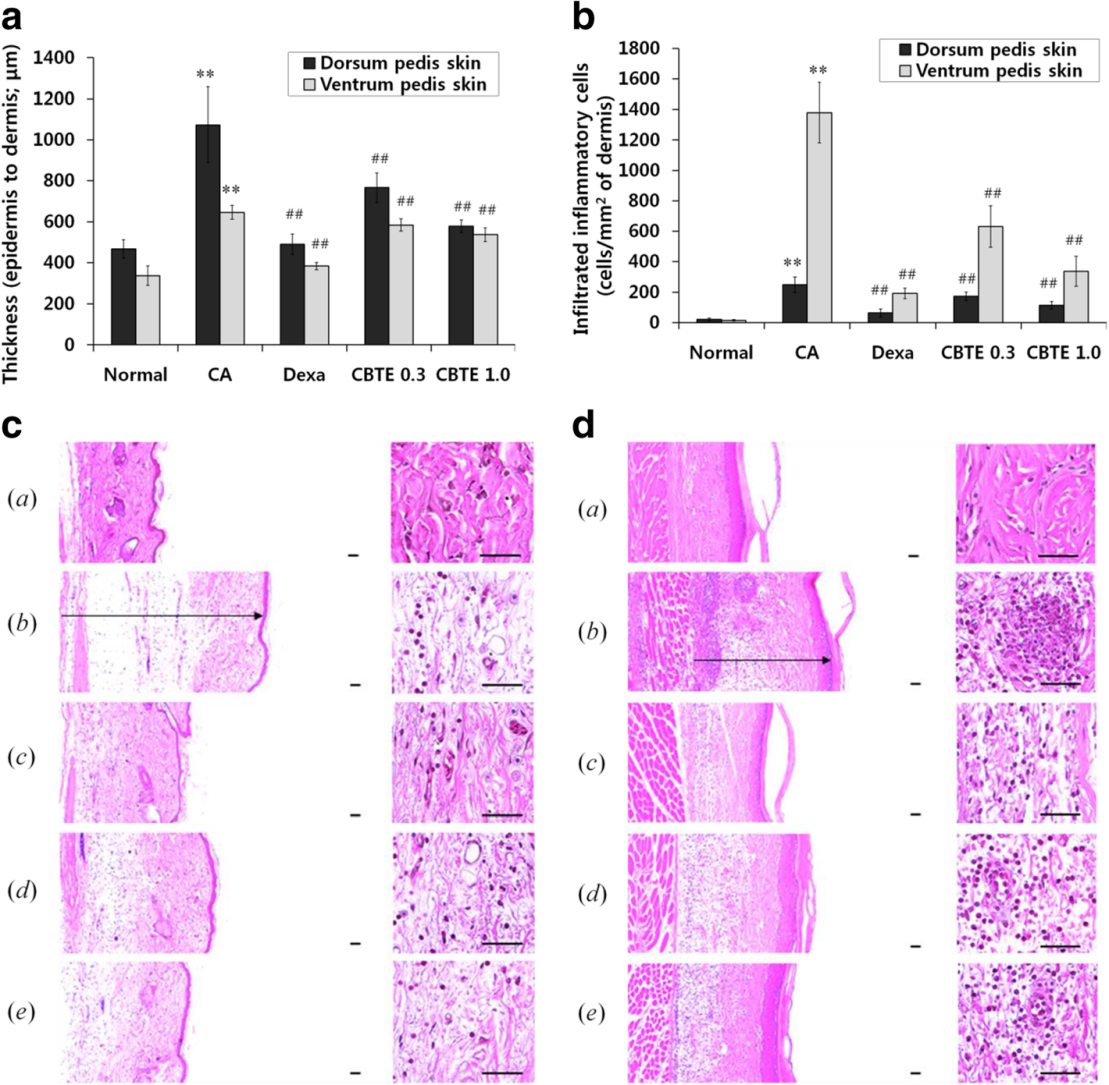
The inhibitory effect of CBTE on paw skin thickness and infiltrated inflammatory cells. Changes in histological profiles, paw skin thickness (**a**) and infiltrated inflammatory cells (**b**) of the dorsum and ventrum pedis skin in normal *a*, carrageenan *b*, dexamethasone *c*, 0.3 g/kg CBTE *d* and 1.0 g/kg CBTE *e* treated groups (**c d**). After 4 h of carrageenan treatment, the dorsum and ventrum pedis skins were separated and fixed in 10% neutral buffered formalin then embedded in paraffin, sectioned and stained with haematoxylin and eosin. Note that marked increases of dorsum (**c**) and ventrum (**d**) pedis skin thicknesses (*arrow*) due to oedematous changes were detected following carrageenan treatment with increases in inflammatory cell infiltrations compared with the normal skin. However, these increases in skin thicknesses and inflammatory cell infiltrations were effectively inhibited by treatment with dexamethasone and were also dose-dependently affected by treatment with two different dosages of CBTE 0.3 and 1.0 g/kg. Scale bars = 60 μm. CBTE, Cheongsangbangpung-tang extract; CA, carrageenan; DEXA, dexamethasone

The effects of CBTE on paw skin thickness and infiltrated inflammatory cells are shown in Fig. 8. The thicknesses of the dorsum pedis and ventrum pedis skins in CA changed by 129.90 and 90.79% compared with normal, respectively. The skin thicknesses of rats treated with DEXA and 0.3 and 1.0 g/kg CBT rats changed by −54.33, −28.53 and −46.16% in the dorsum pedis, respectively, and by −40.57, −9.49 and −16.86% in the ventrum pedis compared with CA. The numbers of infiltrated inflammatory cells in the dorsum pedis and ventrum pedis skins were markedly increased by treatment with CA as compared with normal rat paw skins. However, these CA-induced acute oedematous inflammatory changes were significantly (*P* < 0.01) inhibited by treatment at both different dosages of CBT (0.3 and 1.0 g/kg) in a dose-dependent manner and also in the DEXA treated rats.

**Fig. 9:**
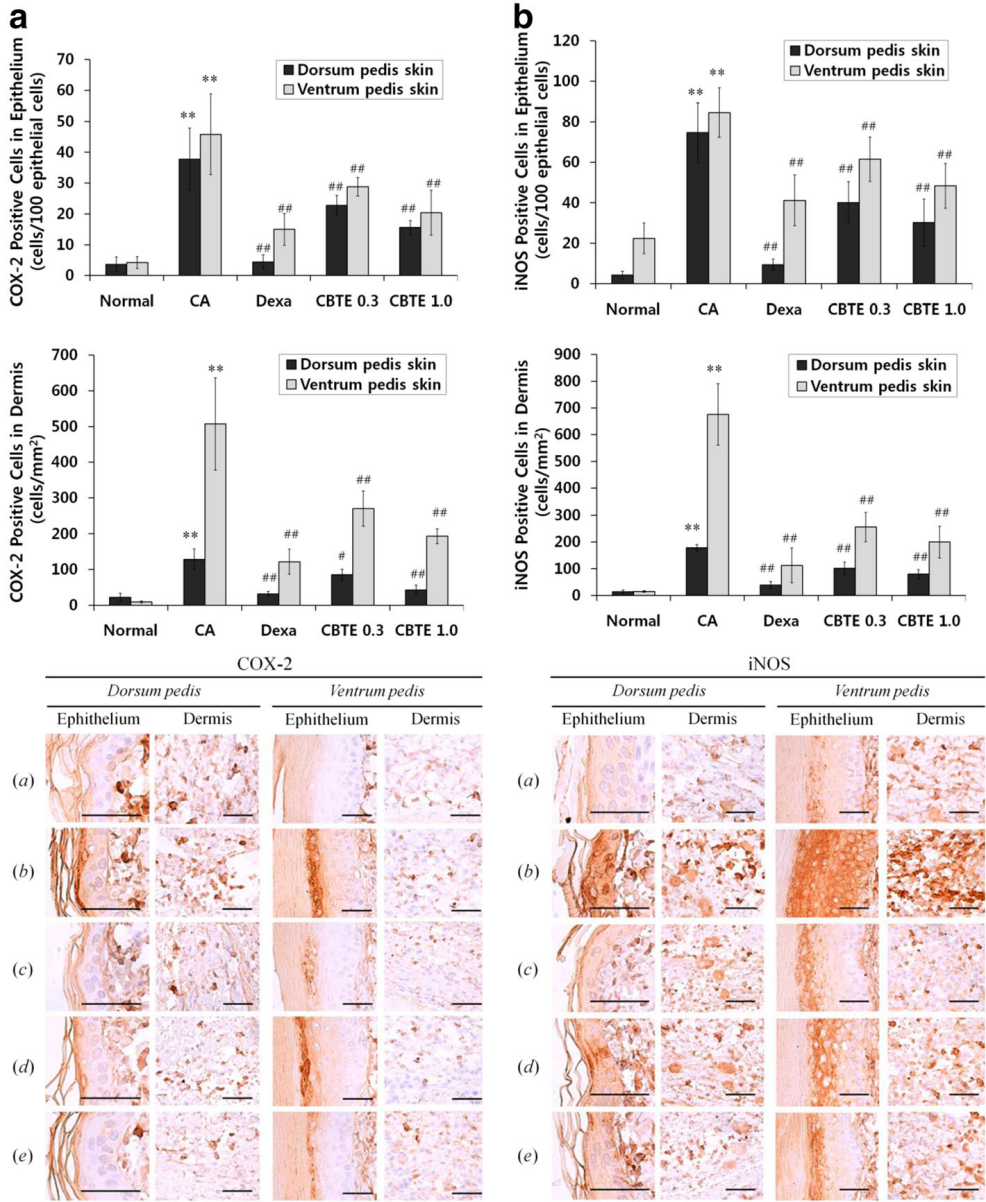
Representative immunohistochemical profiles of COX-2 and iNOS on the paw skins. Note that marked increases of COX-2 (**a**)﻿- and iNOS (**b**)-positive cells were detected on the epithelium and dermis of the dorsum and ventrum pedis skin tissues in CA rats compared with normal rats, respectively. However, these increases of epidermal and dermal COX-2- and iNOS-immunolabelled cells were effectively inhibited by treatment with dexamethasone and were also dose-dependently affected by treatment with CBTE at two different dosages: 0.3 and 1.0 g/kg. Immunoreactive cells were stained using ABC methods where *a* = Normal; *b* = Carrageenan (CA); *c* = CA and dexamethasone; *d* = CA and CBTE 0.3 g/kg; *e* = CA and CBTE 1.0 g/kg. Scale bars = 60 μm. CBTE, Cheongsangbangpung-tang extract; DEXA, dexamethasone

In addition, the representative immunohistochemical profiles of the epithelial and dermis COX-2 and iNOS-immunolabelled cells on the dorsum and ventrum pedis skin are shown in Fig. 9. Marked increases in the epithelial and dermis COX-2 (Fig. 9a) and iNOS-immunoreactive cells (Fig. 9b) were detected in CA. However, these increases were significantly (*P* < 0.01 or *P* < 0.05) inhibited by treatment with both different dosages of CBT and also by DEXA.

## Discussion

Removing heat from the upper body is a characteristic action of CBT in Korean medicine; therefore, CBT has been prescribed in traditional Korean medicine to resolve pimples on the face and neck or to treat eye and ear diseases. The biological activity of CBT has not been extensively studied, although several publications about the antibacterial and anti-acne activity of CBT have produced valid evidence that explains the clinical use of CBT for the treatment of pimples [2, 14]. In the present study, we investigated the effects of CBTE on in vivo and in vitro inflammation. CBTE showed anti-inflammatory properties by regulating markers of inflammation in RAW264.7 murine macrophage treated with LPS and also by relieving CA-induced acute paw swelling in rats.

Inflammation occurs due to infection, physical and chemical injury, ischemia and excessive immune response as part of the host’s attempt to remove damaged tissue and foreign substances, such as invading bacteria [9]. A variety of factors and cells are involved in the inflammatory process. Macrophages are immune cells that primarily trigger the production of inflammatory mediators such as NO and PGE_2_ when activated by bacterial endotoxins like LPS [10, 11]. The increased production of NO and PGE_2_ in LPS-stimulated macrophages can be led by inducible enzymes iNOS and COX-2, respectively [15]. It was reported that the genetic inhibition of iNOS reduced the degree of macrophage inflammation caused by LPS [16]. It has also been shown that selective PGE_2_ synthesis inhibitors can relieve pyrexia and chronic inflammation in rats [17]. We observed that the up-regulated expression of iNOS and COX-2 and the excessive production of NO and PGE_2_ were abolished in the presence of CBTE. Moreover, CBTE had an inhibitory effect on the LPS-induced production of cytokines, such as TNF-α, IL-1β and IL-6, which play important roles in inflammation by producing septic shock syndrome at high levels. TNF-α stimulates the production of interleukins that participate in the response to infection or injury, and IL-1β acts on macrophages to release IL-6 and IL-8 related to the pathogenesis of various inflammatory diseases [18, 19].

NF-κB is a functional transcription factor implicated in inflammation. The activation of NF-κB is critical to induce the expression of various inflammatory enzymes and cytokines, including iNOS, COX-2, TNF-α, IL-1β and IL-6 [20, 21]. In resting macrophages, NF-κB is inactive and placed in the cytosol through interaction with inhibitory protein I-κBα. NF-κB is activated by the phosphorylation and subsequent proteolysis of I-κBα in response to various stimuli, including LPS [22]. We found that LPS potently induced the expression of p-I-κBα, which was restored by CBTE treatment. In line with changes in I-κBα phosphorylation, CBTE inhibited NF-κB activity by decreasing the expression level in the nuclear fraction. These findings suggest that CBTE exerts anti-inflammatory activity through suppressing NF-κB activation in LPS-stimulated RAW264.7 macrophage cells.

Phosphorylation of MAPKs by LPS-stimulation has been shown to initiate inflammatory responses by inducing NF-κB activation [23]. Several reports have indicated that the p38 MAPK pathway is associated with the regulation of inflammatory cytokines [24, 25]. In our study, LPS induced the phosphorylation of three MAPKs (ERK, JNK and p38) that correspond to the increased cytokine level and NF-κB activation. However, CBTE reduced the expression level of p-ERK, p-JNK and p38 protein. These results indicated that the inhibition of MAPK phosphorylation by CBTE was closely associated with the suppression of macrophage inflammation mediated by NF-κB signalling.

Local treatment with CA induces severe oedematous acute inflammations, and a CA-induced hind paw acute oedematous inflammation animal model has been widely used to detect the anti-inflammatory effects of various drugs [26, 27]. Histopathologically, a loosening of connective tissues and inflammatory cell infiltrations mainly involving neutrophils were observed near the CA-treated sites [28, 29]. In the present study, marked rises in the number of infiltrated inflammatory cells with increases of skin thicknesses in both the dorsum and ventrum pedis were detected following treatment with CA as compared with normal rat paw skin. However, these CA-induced acute oedematous inflammatory changes were inhibited by CBTE and also DEXA, respectively. These results are considered direct evidence that CBTE has favourable anti-inflammatory activities.

COX-2, a key enzyme for the synthesis of prostaglandins, which are chemical mediators of inflammation, is also involved in angiogenesis and cancer progression [30, 31]. Therefore, decreases in COX-2 immunoreactivities have been used to predict the favourable effects of test materials on inflammation [32, 33]. In addition, increases in iNOS activities related to proinflammatory agents, such as endotoxin, IL-1β, TNF-α and interferon-γ, can be induced by shock and also occur following inflammatory responses in the body [34]. iNOS is involved in the development of inflammation early after CA administration, and the NO produced by iNOS is responsible for the subsequent maintenance of the inflammatory response. These CA-activated inflammatory cascades are related not only to innate immunity but also to the generation of reactive oxygen species [35]. In the present study, marked increases of epithelia and dermis COX-2 and iNOS-immunoreactive cells were detected on the dorsum pedis and ventrum pedis skin in CA compared with normal rats, which corresponded well with our previous CA rat studies [36, 37]. However, these increases in inflammatory mediator immunolabelled cells were decreased by treatment with both different dosages of CBTE in a dose-dependent manner and also by DEXA. Therefore, CBT should favourably inhibit the acute inflammation mediated by the modulation of inflammatory mediators, such as COX-2 and iNOS expression.

## Conclusions

This study demonstrated the immunoregulatory activity of CBTE via the suppression of transcription factor, NF-κB, which is critical for the expression of cytokines, iNOS and COX-2. In macrophage cells, CBTE decreased the production of LPS-inducible inflammatory mediators, including NO, PGE_2_ and inflammatory cytokines, and inhibited NF-κB activation and MAPK phosphorylation. We also confirmed that CBTE inhibits acute oedematous inflammation through the modulation of COX-2 and iNOS expression in our in vivo study. In conclusion, our results provide scientific evidence to support the clinical efficacy of CBTE in Korean medicine and also contribute to better understanding the molecular mechanism of CBTE in regulating inflammatory responses.

## Abbreviations

CA: Carrageenan
CBT: Cheongsangbangpung-tang
CBTE: Cheongsangbangpung-tang extract
COX: Cyclooxygenase
DEXA: Dexamethasone
ELISA: Enzyme-linked immunesorbent assay
ERK: Extracellular signal-regulated kinase
HE: Hematoxylin & eosin
IL: Interleukin
iNOS: Inducible nitric oxide synthase
JNK: Jun NH_2_-terminal kinase
LPS: Lipopolysaccharide
MAPK: Mitogen-activated protein kinase
MTT: 3-(4,5-dimethylthiazol-2-yl)-2,5-diphenyltetrazolium bromide
NF: Nuclear factor
NO: Nitric oxide
PBS: Phosphate-buffered saline
PG: Prostaglandin
p-I-κBα: Phospho-inhibitor of NF-κB α
RIPA: Radio immunoprecipitation assay
TNF: Tumor necrosis factor
